# Imaging Reactive Oxygen Species with L-012 Reveals Neutrophil Extracellular Trap Formation in Pancreatic Ductal Adenocarcinoma

**DOI:** 10.3390/antiox14121473

**Published:** 2025-12-08

**Authors:** Angisha Basnet, Kaitlyn M. Landreth, Michael Sestito, Kristen Ranson, Seth T. Gammon, David Piwnica-Worms, Brian A. Boone, Tracy W. Liu

**Affiliations:** 1 Department of Microbiology, Immunology, and Cell Biology, West Virginia University, Morgantown, WV 26506, USA; ab00101@mix.wvu.edu (A.B.); kaitlyn.landreth@hsc.wvu.edu (K.M.L.); 2 Division of Surgical Oncology, Department of Surgery, West Virginia University, Morgantown, WV 26506, USA; michael.sestito@hsc.wvu.edu (M.S.); ksranson@hsc.wvu.edu (K.R.); brian.boone@hsc.wvu.edu (B.A.B.); 3 WVU Cancer Institute, West Virginia University, Morgantown, WV 26506, USA; 4 Department of Cancer Systems Imaging, The University of Texas MD Anderson Cancer Center, Houston, TX 77230, USA; stgammon@mdanderson.org (S.T.G.); dpiwnica-worms@mdanderson.org (D.P.-W.)

**Keywords:** reactive oxygen species, neutrophil, neutrophil extracellular traps, pancreatic ductal adenocarcinoma, bioluminescence imaging, L-012, 4-[^18^F]fluoro-1-naphthol ([^18^F]4FN), PET imaging

## Abstract

Neutrophils, key effector cells of the innate immune system, combat pathogens through mechanisms including the production of reactive oxygen species (ROS) and the release of neutrophil extracellular traps (NETs). While these responses are critical for host defense, prolonged elevation of ROS and dysregulated NETosis mediated by neutrophils have been implicated in autoimmune diseases, chronic inflammation, and cancer. In pancreatic ductal adenocarcinoma (PDAC), a highly aggressive and inflammatory malignancy, an increase in neutrophils infiltrating the tumor microenvironment promotes cancer progression and metastasis through increased ROS production and NET release. Using bioluminescence imaging with the reporter L-012 and NET assays, we assessed ROS and NET release, respectively, induced by phorbol myristate acetate and platelet-activating factor in bone-marrow-isolated neutrophils from wild-type and syngeneic myeloperoxidase (MPO)-deficient mice ex vivo. MPO deficiency impaired both ROS generation and NET release, establishing a positive correlation between these processes. In vivo analyses using subcutaneous and spontaneous murine PDAC models revealed elevated ROS in tumors, which were significantly reduced upon genetic deletion of host MPO or peptidyl arginine deiminase 4, an essential enzyme for NET formation, or after treatment with hydroxychloroquine, a NET inhibitor. Furthermore, luminol and 4-[^18^F]fluoro-1-naphthol ([^18^F]4FN), functional L-012 analogs, also enabled non-invasive detection of intratumoral ROS by bioluminescence and PET imaging in vivo, respectively; [^18^F]4FN PET showed a three-fold increased uptake in PDAC tumors versus muscle. PDAC tissues and blood-isolated neutrophils obtained from PDAC patients exhibited elevated ROS compared to controls ex vivo. Importantly, ROS levels correlated strongly with NET formation in patient samples. These findings reveal a bidirectional relationship between ROS and NETs and highlight the potential utility of L-012- and [^18^F]4FN-based PET imaging for monitoring NET-associated inflammation in PDAC in vivo.

## 1. Introduction

Neutrophils are a type of white blood cell that play a crucial role in the body’s innate immune response, serving as the first line of defense against infections, particularly bacterial and fungal pathogens [1,2]. These cells are rapidly recruited to sites of infection and injury, where they perform critical functions to eliminate invading microorganisms through the release of reactive oxygen species (ROS) and the formation of neutrophil extracellular traps (NETs) [1]. While ROS production and NET formation are effective defense mechanisms against pathogens, excessive ROS production and dysregulated NETosis have been implicated in several autoimmune diseases, chronic inflammation, and cancer [3,4,5,6].

When neutrophils are activated, they undergo a process known as the respiratory burst, in which they produce ROS, including superoxide anions, hydrogen peroxide, and hypochlorous acid, through the activation of the nicotinamide adenine dinucleotide phosphate (NADPH) oxidase complex [7,8]. In addition to ROS, neutrophils can engage in a process known as NETosis, where they release extracellular DNA, histones, and antimicrobial proteins to form NETs [1,9]. NETs also contain high concentrations of antimicrobial enzymes, such as myeloperoxidase and NADPH oxidase, to enhance their pathogen-killing abilities [9,10]. These two pathways, ROS and NET production, are crucial indicators of neutrophil activation.

Dysregulated neutrophil activation pathways are increasingly recognized as contributors to cancer progression, highlighting the importance of monitoring these processes for early detection and disease management. However, monitoring highly reactive short-lived ROS production and NET formation is technically challenging. A few studies have shown that NET formation is thought to rely on ROS generated by NADPH oxidase and myeloperoxidase (MPO) activity, but little is known regarding the link between these two interconnected neutrophil activation pathways [11,12,13]. During cancer progression, especially in pancreatic ductal adenocarcinoma (PDAC), neutrophils comprise a substantial amount of the immune infiltrate within the tumor microenvironment [14,15]. Elevated NETs and ROS levels have been shown to correlate with PDAC progression, metastasis, treatment resistance, and poor patient outcomes [16,17,18,19]. Thus, understanding the interplay between ROS and NETs could provide insights into the mechanistic links between these neutrophil activation pathways, while also guiding the development of more effective strategies for monitoring these pathways.

Several techniques have been developed to study ROS, including the use of fluorescent probes such as 2′,7′-dichlorodihydrofluorescein, cytochrome c reduction assays, and flow cytometry [20,21,22]. Among them, bioluminescence-based methods utilizing the reporter molecules luminol and L-012 (8-amino-5-chloro-7-phenylpyrrido [3,4-d]-pyridazine-1, 4 (2H, 3H) dione) have emerged as highly sensitive tools for the detection of NADPH-derived ROS [23,24]. While luminol enables MPO-specific ROS detection in vivo, L-012, a luminol-based probe, emits enhanced light upon oxidation by ROS [23,25,26,27]. L-012 is particularly advantageous because it can detect low levels of ROS and provides a more sustained signal than other luminescent probes, such as luminol and lucigenin, making L-012 ideal for long-term bioluminescence imaging as demonstrated in monitoring inflammatory responses [25,26,28,29]. Importantly, this method allows researchers to non-invasively monitor ROS production in real-time.

In this study, we demonstrate a strong positive correlation between neutrophil ROS and NET levels using in vitro assays, preclinical PDAC models, and human PDAC samples. Our findings establish L-012–based ROS detection and related agents as robust tools for in vivo tracking of dynamic inflammatory responses in cancer and highlight their potential utility as surrogate markers of NETosis.

## 2. Materials and Methods

### 2.1. Reagents

Luminol (sodium salt), platelet-activating factor-16 (PAF), DNase and 2-(4-Carboxyphenyl)-4,4,5,5-tetramethylimidazoline-1-oxyl-3-oxide (CPTIO) were purchased from Sigma-Aldrich (Sigma-Aldrich, St. Louis, MO, USA). L-012 sodium salt was purchased from Wako Chemicals USA (Wako Chemicals USA, Inc., Richmond, VA, USA). Luminol sodium salt was dissolved in sterile phosphate-buffered saline (PBS) to a final concentration of 50 mg/mL and stored at −20 °C. L-012 powder was dissolved in sterile double-distilled water (ddH20) to a final concentration of 20 mM and stored at −20 °C. Quant-iT™ PicoGreen™ dsDNA assay kits, phorbol 12-myristate 13-acetate (PMA), Gibco Poly-D-Lysine, Hydroxychloroquine sulfate (HCQ) and Sytox™ Deep Red Nucleic Acid Stain were purchased from Thermo Fisher Scientific (Thermo Fisher Scientific, Waltham, CA, USA). PMA is dissolved in DMSO to a final concentration of 1 mg/mL and stored at −20 °C. PAF is dissolve in ddH20 to a final concentration of 1 mM and stored at −20 °C. Sytox™ Deep Red Nucleic Acid Stain was dissolved in PBS to make a final concentration of 1 mM before storing at −20 °C. Stock solution of DNase dissolved in PBS at a concentration of 10 mg/mL. All-trans retinoic acid (ATRA), diphenyleneiodonium (DPI), and verdiperstat (Verd, AZD5904) were purchased from MedChemExpress (MedChemExpress LLC, Princeton, NJ, USA).

### 2.2. In Vivo Tumor Model

The Institutional Animal Care and Use Committee at West Virginia University approved all animal protocols (2109047227). KPCY6419c5 (KPCY6419) cells were purchased from Kerafast (Kerafast, Boston, MA, USA). 2 × 10^5^ KPYC6419 cells were injected subcutaneously on the right flank of 8-week-old female C57BL/6 wild-type, WT (The Jackson Laboratory, Bar Harbor, ME, USA), age matched syngeneic C57BL/6 myeloperoxidase-deficient, *MPO^−/−^* (B6.129X1-Mpo^tm1Lus^/J, The Jackson Laboratory, ME, USA) or age matched syngeneic C57BL/6 peptidylarginine deiminase 4-deficient, *PAD4^−/−^* (B6.Cg-Padi4^tm1.1Kmow^/J, The Jackson Laboratory, ME, USA) mice. KPCY6419 cells were cultured in DMEM supplemented with 10% heat-inactivated fetal bovine serum and 1% glutamine, maintained at 37 °C in a humidified atmosphere containing 5% CO_2_. Prior to tumor cell injection, the fur was removed on the right flank. Tumors were measured using calipers twice weekly and tumor volume was calculated using the formula 0.5× (Length × Width^2^). For the spontaneous PDAC model (KPC, Kras^G12D/+^; Trp53^R172H/+^; Pdx-1-Cre, The Jackson Laboratory, ME, USA), mice were monitored weekly by abdominal palpation for spontaneous tumor growth. The KPC mice were generated by breeding KP mice (B6.129-Kras^tm4Tyj^ Trp53^tm1Brn^/J, The Jackson Laboratory, Bar Harbor, ME, USA) and Pdx-Cre mice (B6.FVB-Tg(Pdx1-cre)6Tuv/J, The Jackson Laboratory, Bar Harbor, ME, USA).

### 2.3. Quantification of ROS Levels

Neutrophils were isolated from the bone marrow of healthy male WT and *MPO^−/−^* mice using an EasySep™ mouse neutrophil enrichment kit (STEMCELL Technologies Inc., Cambridge, MA, USA). Gr1^+^ myeloid cells were isolated from the spleen of tumor-bearing spontaneous KPC mice at endpoint (week 12–17) using EasySep™ mouse MDSC (CD11b^+^Gr1^+^) isolation kit (STEMCELL Technologies Inc., Cambridge, MA, USA). A total of 1 × 10^5^ isolated cells were added to 96 well black-walled plate in 100 μL phenol-free DMEM media and immediately imaged for 60 min with 50 mM luminol or 10 μM L-012. To activate bone-marrow-isolated neutrophils, 500 nM PMA or 50 μM PAF was added to respective wells immediately prior to imaging them on the Kino imaging system (Spectral Instruments, Tucson, AZ, USA). Typical acquisition parameters are as follows: acquisition time, 5 min; binning, 16; FOV, 13 cm; f/stop, 1.2; filter, open; total number of acquisitions 12; 5% CO_2_ flow. Bioluminescence photon flux (photons/s) data were analyzed by ROI measurements in Aura imaging software (version 4.5.0) (Spectral Instruments, Tuscon, AZ, USA). Raw data were imported into Microsoft Excel (Microsoft Corp., Redmond, WA, USA) and further analyzed using GraphPad Prism (version 10.2.3, GraphPad Software Inc., La Jolla, CA, USA) where the highest photon flux within the 60-min imaging window was identified and plotted.

For the L-012 and luminol specificity assay, HL60 cells were differentiated into a ‘neutrophil-like’ phenotype using 1 μM ATRA for 3 days in culture [30]. To induce ROS production, cells were stimulated with 500 nM PMA. Following PMA stimulation, cells were immediately treated with specific inhibitors: 10 μM DPI, 500 μM Verd, 100 μM CPTIO. The impact of each inhibitor on ROS production was assessed using 50 mM luminol or 10 μM L-012 bioluminescence imaging on the Kino system using the same parameters used for imaging murine neutrophils as described above.

### 2.4. In Vivo IVIS Imaging

MPO activity and ROS level were measured in vivo in WT, *MPO^−/−^* and *PAD4^−/−^* mice at day 17 post-tumor cell inoculation when subcutaneous tumors reached a diameter of ≥ 5 mm. For spontaneous tumor KPC mice, L-012/luminol bioluminescence imaging began between week 9–10 and continued weekly until the experimental endpoint, defined by criteria including ≥20% body weight loss, lethargy, larger ascites, or neurological symptoms. Healthy (tumor-free) WT mice were used as controls. To visualize MPO and ROS levels, mice were intraperitoneally injected with either luminol (200 mg/kg body weight) or L-012 (25 mg/kg body weight). Images were acquired 9 min post bioluminescence substrate injection using the IVIS spectrum (Perkin Elmer, Waltham, MA, USA); typical acquisition parameters as follows: acquisition time, 5 min auto; binning, 8; FOV, 22.6; f/stop, 1; filter, open. For HCQ studies, fresh HCQ solution was prepared in ddH20 at a concentration of 12.5 mg/mL and 200 μL was injected intraperitoneally at day 14 and 17 post-tumor injection. For DNase treatment, 250 μL of 0.5 mg/mL of stock DNase was injected intraperitoneally at day 14 and day 22 post-tumor injection. L-012 and luminol bioluminescence imaging were performed at baseline (0 h) and 24 h post-treatment using the same acquisition parameters described above. Quantifying the bioluminescence imaging data occurred using the Living Image software (version 4.5.2, Perkin Elmer, Waltham, MA, USA). A background ROI was drawn for each image to measure the background bioluminescence. The bioluminescence within the tumor of individual mice was quantified by drawing a uniform background-subtracted ROI around the tumor (right flank for subcutaneous tumor and abdomen for spontaneous KPC mice). The total flux obtained from the post-treatment (24 h) image was normalized to the pre-treatment total flux (0 h) image for each mouse in Microsoft Excel and plotted as a fold-change value in GraphPad Prism.

### 2.5. NETs Quantification

NETs were quantified using a PicoGreen dsDNA reagent kit (Thermo Fisher Scientific, Waltham, CA, USA) following the previous protocol with slight modifications [31]. 1 × 10^5^ mouse bone-marrow- or PDAC patient blood-isolated neutrophils were suspended in 100 μL of DMEM and incubated for 1–2 h in a 96-well plate. PicoGreen fluorescence (Excitation: 485/20 nm and Emission: 528/20 nm) was measured using the Synergy HTX multi-mode plate reader (BioTek Instruments, Winooski, WT, USA). The DNA concentration of the sample was determined using the lambda DNA standard curve plotted in Microsoft Excel.

### 2.6. Sytox Red Imaging

1 × 10^5^ bone-marrow-isolated neutrophils in 100 μL phenol-free DMEM media were added to poly-D-Lysine coated 96-well plate. 500 nM PMA was added and incubated for 2 h prior to imaging using the Lionheart imaging system (BioTek Instruments, Winooski, WT, USA). Sytox deep red stain (1 μM) was added immediately before imaging. Representative images were taken at 20× objective.

### 2.7. Human PDAC Sample

Patients diagnosed with PDAC were identified and consented for blood procurement (IRB #2502110227 and #1903496995). Blood samples were obtained immediately prior to surgery, following completion of neoadjuvant chemotherapy or neoadjuvant chemotherapy combined with HCQ (Clinical trial: NCT04911816) as detailed in Table 1. Blood from patients with intraductal papillary mucinous neoplasms (IPMN) was used as a non-cancer sample control. Human blood neutrophils were isolated using a MACSxpress Whole Blood Neutrophil Isolation Kit according to the manufacturer’s protocol (Miltenyi Biotec, Waltham, MA, USA). 1 × 10^5^ neutrophils were added to 10 μM L-012 in 100 μL PBS in a 96-well black-walled plate. Neutrophils were imaged and analyzed following the previously published protocol with the same acquisition parameters [32].

Freshly obtained human PDAC and adjacent non-tumor tissue were placed in a 12-well plate with 20 mM L-012 and immediately imaged for 60 min using the Kino imaging system (same acquisition parameters as blood-isolated neutrophils). For each sample, the highest photon flux observed during the 60-min imaging period was identified and plotted, using the same analysis approach described above for L-012 imaging of mouse neutrophils.

### 2.8. PET (Positron Emission Tomography) Imaging

All PET imaging experiment were conducted under the Institutional Animal Care and Use Committee protocol 0001179-RN03, approved by the University of Texas MD Anderson. At 11 weeks of age, 2 × 10^5^ KPCY6149 cells in 100 μL PBS were injected to right flank of 5 mice. Three weeks post-tumor implantation (14 weeks old), mice were imaged by PET/CT with [^18^F]4FN. [^18^F]4FN was produced at pre-clinical scales as previously published [30]. Mice (n = 5) were injected intravenously with 90 uCi of [^18^F]4FN and were allowed to eat and drink for 1 h. Mice were then lightly anesthetized and imaged using an Albira PET/CT imaging system (Bruker, Billerica, MA, USA) following established protocols [30]. Briefly, a 10-min PET acquisition was performed, followed by a single CT series encompassing the tumor and abdomen. Image reconstruction was conducted as previously described [30]. Volumes of interest (VOI) drawn around tumors, heart, and contralateral muscle used the PMOD quantification software (version 4.5, Bruker, Billerica, MA, USA) using the CT data as a guide. Ratios of mean activity concentration in the VOI from PET data were calculated on a per mouse basis.

### 2.9. CiH3 (Citrullinated Histone H3) ELISA

Human serum was collected through centrifugation of human blood at 1000 rpm for 10 min. Following collection, serum was aliquoted and stored at −80 °C until analysis. Prior to assay, samples were thawed and diluted to 1:2. CitH3 levels were quantified using the Cayman Chemical Citrullinated Histone H3 ELISA kit (Clone 11D3, Ann Arbor, MI, USA), following the manufacturer’s protocol and a previously published method [33]. Absorbance was measured at 450 nm, and CitH3 concentrations were determined from a standard curve.

### 2.10. Statistical Analysis

Graphs were made and statistical analyses were performed using GraphPad Prism (GraphPad Software, Inc, La, Jolla, CA, USA). Data are expressed as the mean ± SD. Analysis of differences between two groups were performed using an unpaired Student’s t-test. For analysis of three or more groups, analysis of variance (ANOVA) tests was performed. *p* values were considered statistically significant if *p* < 0.05.

## 3. Results

### 3.1. Increased ROS Correlates with NET Levels

L-012 has been used previously as a measure of ROS levels [34,35]. To evaluate the specificity of L-012 for different ROS generated via respiratory burst pathways, PMA-stimulated HL60 cells were incubated with the following ROS pathway inhibitors: DPI, a NADPH oxidase (NOX) inhibitor; Verd, a MPO inhibitor, and CPTIO, a NO scavenger (Figure S1a). Since NOX and MPO are the primary sources of ROS in neutrophils (Figure S1a), inhibition of either the NOX or MPO pathways significantly suppressed ROS production (Figure S1b,c). L-012 bioluminescence was reduced 23.25 ± 4.80-fold with NOX inhibition and 162.28 ± 16.42-fold with MPO inhibition. Treatment with CPTIO also resulted in a moderate reduction in ROS levels, demonstrating a decrease in L-012 bioluminescence of 3.91 ± 0.56-fold compared to the PMA-stimulated control (Figure S1b,c). These data suggest that in PMA-stimulated neutrophils, L-012 predominantly detects MPO-derived ROS.

**Figure 1:**
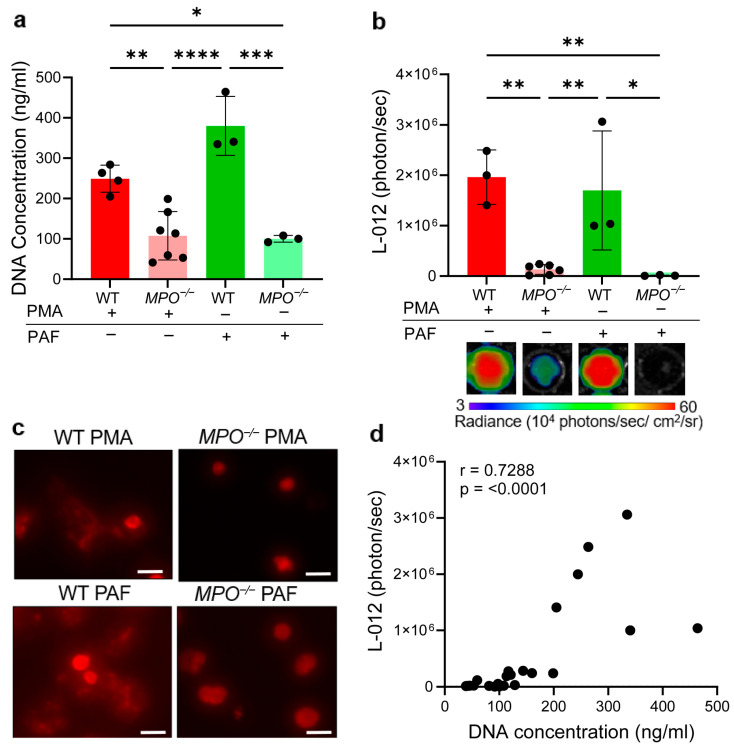
Correlation of neutrophil ROS with NET levels. (**a**) Quantification of NETs using a PicoGreen assay. (**b**) Quantification of ROS production and corresponding representative images of L-012 bioluminescence. (**c**) Representative Sytox deep red fluorescence images showing extracellular DNA release in neutrophils isolated from WT and *MPO^−/−^* mice, stimulated with PMA and PAF; scale bar 10 µm. (**d**) Correlation analysis between L-012 bioluminescence and NET levels quantified using PicoGreen. Data are representative of n = 3–7 independent experiments and shown as the mean ± SD; each black dot corresponds to neutrophils isolated from a single mouse. Statistical significance was determined using one-way ANOVA followed by Tukey’s multiple comparison test, * *p* < 0.05, ** *p* < 0.01, *** *p* < 0.001, **** *p* < 0.0001. Correlation analysis was performed using Pearson’s correlation coefficient (r), 95% confidence interval [0.46, 0.87].

Given the MPO dependence of L-012 bioluminescence, we evaluated NET formation and ROS production in primary neutrophils isolated from the bone marrow of WT and *MPO^−/−^* mice stimulated with PMA and PAF, which are known inducers of both responses. A significant increase in NET formation and ROS level in WT neutrophils was observed following simulation with both PMA and PAF compared to *MPO^−/−^* neutrophils (Figure 1a,b). Even under unstimulated conditions, basal NET and ROS levels were markedly higher in WT neutrophils compared to *MPO^−/−^* neutrophils (Figure S1d,e). NET formation was further validated using Sytox deep red staining, which revealed a characteristic morphological transition in stimulated WT neutrophils of extracellular DNA spread, indicative of NET release which was not observed in *MPO^−/−^* neutrophils (Figure 1c). Additionally, a strong positive correlation was observed between ROS and NET levels (Figure 1d). To capture the full spectrum of neutrophil activation states, correlation analyses included datasets from unstimulated and PMA- or PAF-stimulated neutrophils from both WT and *MPO^−/−^* mice. The wide distribution of values, particularly in the double low region, reflects expected biological heterogeneity in minimally activated or MPO-deficient neutrophils which exhibit low ROS levels and minimal NET release. Importantly, this distribution indicates that the association between ROS and NET formation holds across both low and high neutrophil activation states. These findings indicate that increased ROS production in neutrophils is strongly associated with enhanced NET formation.

To further validate the L-012 data, luminol imaging was employed as a secondary method to measure MPO-dependent ROS production (Figure S2a,b). The specificity of luminol for MPO activity was confirmed by pretreating PMA-stimulated HL60 cells with DPI, verd, or CPTIO. As expected, DPI and verd significantly suppressed luminol signals (luminol bioluminescence was reduced 13.52 ± 10.93-fold with DPI and 23.08 ± 26.13-fold with verd), while CPTIO had no significant effect (fold-change of 2.37 ± 1.22), consistent with luminol’s specificity for MPO-derived ROS (Figure S2a,b). Following PMA and PAF stimulation, WT neutrophils exhibited a pronounced increase in MPO activity compared to *MPO^−/−^* neutrophils quantified using luminol (Figure S2c). When comparing unstimulated neutrophils isolated from WT and *MPO^−/−^* mice, no significant difference in basal luminol signal was observed (Figure S2d). However, while overall signal was lower, an even stronger positive correlation between MPO activity and NET levels was observed (Figure S2e), consistent with the known specificity of luminol for MPO-mediated ROS release in living cells [24,30]

### 3.2. Elevated ROS Levels in Murine PDAC Models In Vivo

**Figure 2:**
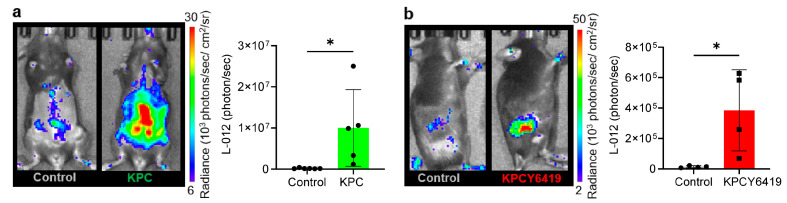
In vivo ROS production in murine PDAC models using L-012. Representative images and quantification of L-012 bioluminescence in (**a**) spontaneous KPC (week 12–17) and (**b**) subcutaneous WT KPCY6419 PDAC tumors at endpoint (day 24 post tumor cell injection) compared to healthy WT tumor-free controls. Data are shown as the mean ± SD; n = 4–6 mice/group; each black dot or square corresponds to a measurement from a single mouse. Statistical analysis was performed using an unpaired student *t*-test, * *p* < 0.05.

To assess intratumoral ROS levels during pancreatic tumor progression, we performed in vivo L-012 bioluminescence imaging in both spontaneous KPC and subcutaneous murine models of PDAC. In the spontaneous KPC PDAC model, significantly elevated ROS signals were observed at the endpoint (weeks 12–17) compared to tumor-free control WT mice (Figure 2a). Similarly, subcutaneous PDAC tumor-bearing mice displayed increased intratumoral ROS levels at day 24 post-tumor implantation relative to healthy (tumor-free) WT controls (Figure 2b). These findings indicate that PDAC progression is associated with increased ROS levels in vivo detectable using L-012.

To validate these findings, we utilized luminol imaging, which also revealed a significant increase in MPO-dependent ROS levels in both the spontaneous KPC and subcutaneous PDAC tumors (Figure S3a,b). In addition, analysis of Gr1^+^ myeloid cells isolated from the spleens of KPC mice showed significantly enhanced basal levels of NETs and ROS at tumor endpoint compared to healthy mice (Figure S4a,b). These findings were consistent with our previous work demonstrating increased NET levels and ROS production in Gr1^+^ splenic-derived myeloid cells isolated from subcutaneous PDAC tumor-bearing mice [31]. In the present study, we extended this analysis by performing correlation studies, which revealed a strong positive association between ROS and NET levels (Figure S4c), further supporting the link between tumor-associated neutrophil ROS and NET levels during PDAC progression.

### 3.3. Inhibiting NETs Also Reduces ROS Levels

**Figure 3:**
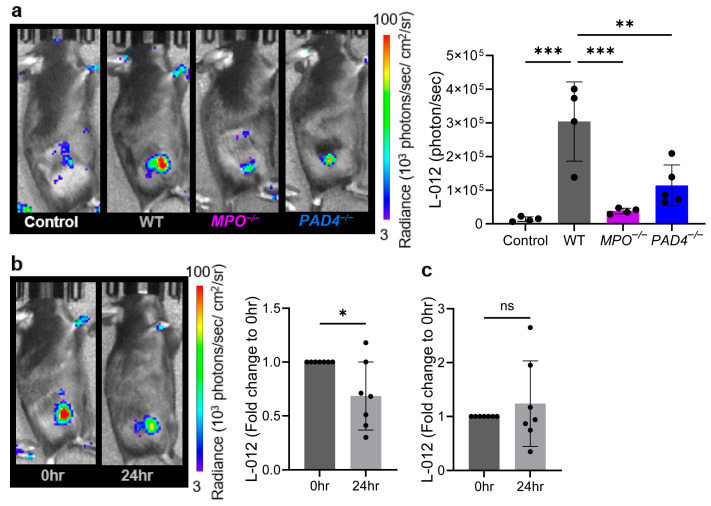
In vivo inhibition of NETs decreases ROS levels. Representative bioluminescence images and corresponding L-012 quantification of ROS levels in (**a**) healthy WT tumor-free controls and WT, *MPO^−/−^*, *PAD4^−/−^* subcutaneous KPCY6419 tumor-bearing mice at days 17 post tumor cell injection (control mice are the same dataset from Figure 2b), (**b**) pre- (0 h) and 24 h post (24 h) HCQ treatment at days 14 and 17 post-tumor injection and (**c**) pre (0 h) and 24 h post (24 h) DNase treatment at days 14 and 22 post tumor injection in KPYC6419 tumor-bearing WT mice. WT data are shown in panel a (WT) and panel b (0 h) are derived from the same cohort of mice at day 17 post tumor injection. Data are shown as the mean ± SD; n = 4–7 mice/group; each black dot corresponds to a measurement from a single mouse. Statistical analysis was performed using one-way ANOVA followed by Tukey’s multiple comparison test and unpaired student *t*-test, * *p* < 0.05, ** *p* < 0.01, *** *p* < 0.001, ns = non signifiticant.

To investigate the contribution of ROS in NET formation, we assessed intratumoral ROS levels in *MPO^−/−^*, *PAD4^−/−^*, and WT mice using the subcutaneous KPCY6419 PDAC model. MPO-deficient mice are known to have a defective MPO-derived ROS production in myeloid cells, while PAD4-deficient mice exhibit impaired NET formation [36,37,38]. Intratumoral ROS levels measured using L-012 were significantly reduced in *MPO^−/−^* mice, showing an 8.27 ± 1.88-fold decrease compared to WT mice (Figure 3a), indicating that most of L-012-detectable ROS in PDAC tumors is MPO-dependent. Interestingly, ROS levels were also significantly decreased in *PAD4^−/−^* mice, showing a 3.21± 1.36-fold reduction in L-012 bioluminescence compared to WT mice (Figure 3a). The ROS levels in both *MPO^−/−^* and *PAD4^−/−^* mice were similar and not significantly different from basal levels observed in tumor-free WT mice (Figure 3a), suggesting that L-012 bioluminescence in this PDAC model primarily reflects NET formation. To further explore this, we treated WT mice with HCQ which has been shown to inhibit NETs [33,39]. 24 h following HCQ treatment, ROS levels in the tumors were significantly lower compared to pre-treatment levels (Figure 3b). In contrast, treatment with DNase, which degrades NETs after their formation, did not affect intratumoral ROS levels after 24 h (Figure 3c). This was further evaluated using luminol to assess the contribution of MPO-specific ROS to NETs. Consistent with the L-012 measurements, both *MPO^−/−^* and *PAD4^−/−^* tumors showed reduced MPO activity (fold reduction of 4.09 ± 1.16 and 4.43 ± 1.9, respectively) compared to the WT control (Figure S5a). Moreover, MPO activity in these *MPO^−/−^* and *PAD4^−/−^* mice was similar to levels observed in tumor-free WT mice, further supporting that NETs are a major source of intratumoral MPO-specific ROS. Similarly, HCQ-treated tumors displayed a significant reduction in MPO activity 24 h post-treatment, mirroring L-012 data (Figure S5b). However, DNase treatment resulted in increased MPO activity post-treatment compared to pre-treatment levels (Figure S5c). These findings collectively demonstrate that impaired NET formation, whether due to PAD4 deficiency or HCQ treatment, resulted in reduced ROS production within the tumor microenvironment, while DNase-mediated NET degradation did not impact ROS levels.

### 3.4. PET Imaging

**Figure 4:**
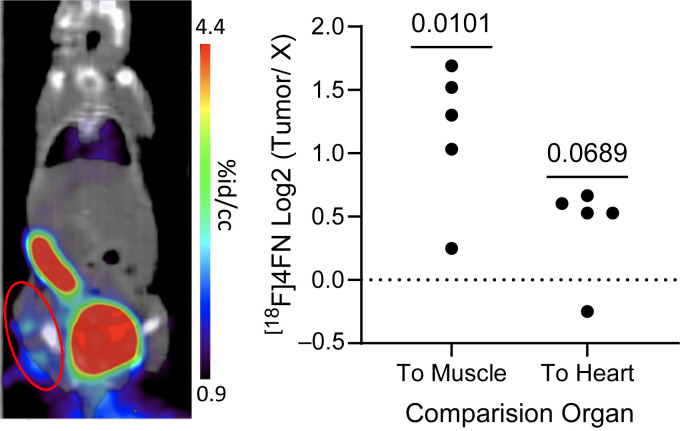
Non-invasive intratumoral ROS imaging using [^18^F]4FN PET. Representative coronal PET/CT image, where the red circle highlights subcutaneous tumor, and corresponding quantification of PET scan signals after intravenous injection of [^18^F]4FN in subcutaneous KPCY6419 tumor-bearing WT mice (n = 5 mice, each black dot corresponds to a measurement from a single mouse.). High-intensity signals in the urinary bladder and intestinal lumen/fecal material reflect normal renal and hepatobiliary clearance pathways for 4FN [30], respectively.

While L-012 bioluminescence has been a widely used tool for detecting ROS in preclinical animal models, its translational potential for clinical in vivo imaging remains limited. The primary challenges include low signal intensity and limited tissue penetration in human subjects, which constrain its utility for detecting ROS in deeper anatomical sites [40]. Given these limitations, we transitioned to PET imaging using [^18^F]4FN, a functional analog of L-012, for non-invasive detection of intratumoral ROS in the subcutaneous KPCY6419 PDAC model [30]. In our study, PET imaging revealed a three-fold increase of [^18^F]4FN uptake in the tumor compared to muscle tissue (Figure 4), demonstrating high sensitivity and specificity for in vivo intratumoral ROS detection. It is important to note that [^18^F]4FN PET provides one snapshot in time, while L-012 is capable of dynamic imaging over time. Nonetheless, given the observed correlation between L-012 ROS and NET formation in Figure 3, [^18^F]4FN PET imaging holds promise as a clinically applicable tool for detecting intratumoral ROS and potentially NET levels.

### 3.5. Elevated ROS Levels in PDAC Patient Samples Correlate with Increased NETs

**Figure 5:**
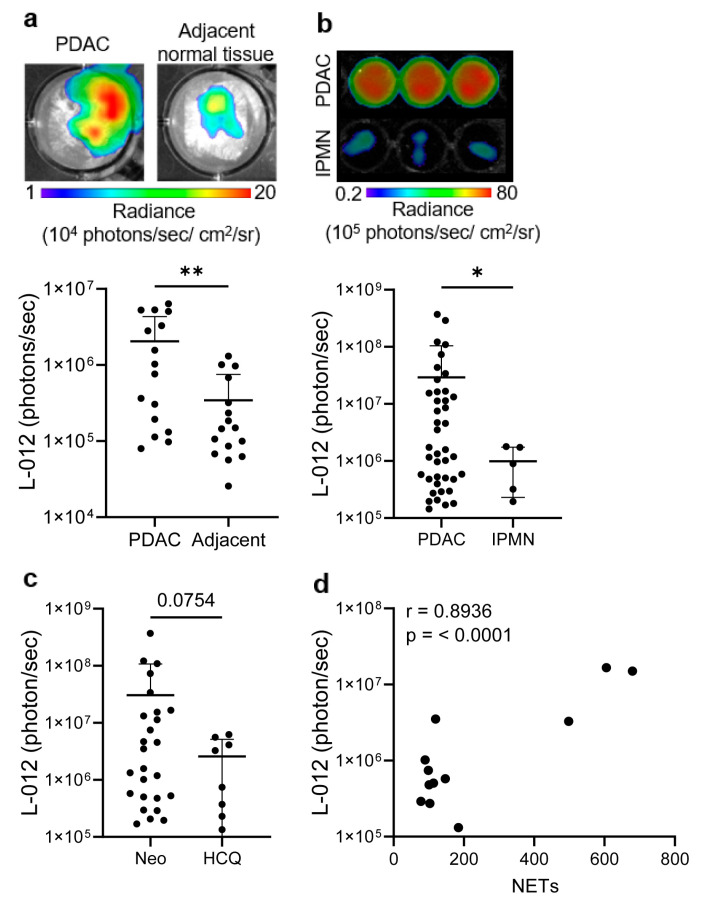
Elevated ROS levels in PDAC patient samples correlate with increased NETs. (**a**) Representative bioluminescence images and corresponding L-012 quantification of ROS levels in PDAC patient tumor vs. adjacent normal tissue (n = 16 patients/group). (**b**) Representative bioluminescence images and corresponding L-012 quantification of ROS level in blood-isolated neutrophils from (**b**) PDAC (n = 41 patients, excluding HCQ-treated group as listed in Table 1) compared to IPMN (n = 5 patients), and (**c**) neoadjuvant-treated (Neo, n = 26 patients) compared to HCQ-treated (n = 8 patients) PDAC patients. (**d**) Correlation analysis between L-012 bioluminescence signal (ROS levels) and NETs measured by PicoGreen in blood-isolated neutrophils from PDAC patients (n = 12 patient neutrophils). Data are shown as the mean ± SD on a logarithmic *y*-axis where each black dot represents an individual patient.; unpaired student *t*-test, * *p* < 0.05, ** *p* < 0.01. Correlation analysis was performed using Pearson’s correlation coefficient (r), 95% confidence interval [0.66, 0.97].

We further investigated ROS levels ex vivo using PDAC patient tissue and blood-isolated neutrophils. We observed a significant increase in ROS level in PDAC tissue compared to adjacent non-cancerous tissue when incubated with L-012 (Figure 5a). Similarly, blood-isolated neutrophils from PDAC patients showed significantly increased ROS when compared to neutrophils from patients with IPMN (non-PDAC) measured using L-012 bioluminescence (Figure 5b). In patients treated with HCQ, a downward trend in ROS levels was observed in blood circulating neutrophils compared to the neoadjuvant treatment group (Figure 5c). Moreover, serum ELISA measurement of citH3, a specific marker of NET formation, demonstrated a significant decrease in serum citH3 level in patients treated with HCQ compared to the neoadjuvant group (Figure S6), similar to what was observed in a previous study [33]. However, as a circulating biomarker, serum citH3 reflects systemic NET burden and may be influenced by factors beyond neutrophil intrinsic NET-forming capacity, such as circulating DNA. To directly assess NET formation from neutrophils themselves, we performed a PicoGreen assay on the supernatant from blood-isolated neutrophils of PDAC patients. This assay quantifies extracellular DNA released by neutrophils alone [41,42]. The result demonstrated a strong positive correlation between NET and ROS levels in these neutrophils (Figure 5d). These clinical observations mirror our preclinical data, further reinforcing the link between ROS and NETosis in human PDAC.

## 4. Discussions

Inflammation is a well-recognized hallmark of cancer, playing a complex role that can either suppress tumor growth or promote tumor progression when dysregulated [43,44]. Neutrophils, a key innate immune cell, are critical effectors in this inflammatory milieu, and contribute to pancreatic tumor inflammation by being actively recruited to the tumor microenvironment [14,45]. Emerging evidence highlights NETs as critical drivers of PDAC progression by immune modulation and promoting tumor cell proliferation and metastasis [46,47]. ROS are also implicated in various cellular processes, including proliferation, growth, apoptosis, and invasion, as well as contributing to the tumor microenvironment [16,17]. Both the NET and ROS pathways have been explored as therapeutic targets in multiple cancers, including PDAC. However, the lack of reliable, non-invasive imaging modalities to monitor ROS- and/or NET-driven inflammation in vivo limits the ability to rigorously assess therapeutic interventions targeting these pathways. Herein, results connect neutrophil ROS levels to NETs and demonstrate that imaging with L-012 and [^18^F]4FN can capture ROS-driven inflammatory dynamics in PDAC.

Neutrophils were activated by two distinct stimuli, PMA and PAF, to understand the correlation of ROS level and NET formation in vitro. PMA results in neutrophil activation by stimulating protein kinase C, initiating a cascade of events that lead to ROS production, degranulation, and NETosis [48]. In contrast, PAF is a phospholipid that activates neutrophils through a surface receptor and is considered a weaker stimulus of oxidative burst but a strong NET inducer [49,50]. Neutrophil activation with PAF induced a rapid but transient burst of ROS production (peaking within 5 min of exposure), whereas PMA-stimulated neutrophils exhibited a delayed ROS response, beginning 10–15 min post-stimulation that persisted for >60 min. These findings align with previous studies using bovine neutrophils stimulated with PAF and PMA [51]. MPO-deficient neutrophils exhibited a significant reduction in ROS and NET induction following PMA and PAF stimulation compared to WT neutrophils (Figure 1a–d). There was a strong correlation between ROS and NETosis, further validated using the MPO-specific luminol reporter (Figure S2). These data support MPO as a key ROS-generating enzyme in neutrophils isolated from C57BL/6 mice. To further confirm the ROS specificity of L-012, HL60 cells, a widely used human leukemia cell line that can differentiate into neutrophil-like cells, were stimulated with PMA [30,52]. Pharmacologic inhibition of MPO in PMA-stimulated HL60 cells significantly reduced L-012 bioluminescence compared to PMA alone. This indicates that MPO-derived HOCl contributes to neutrophil L-012 specificity, consistent with previous studies [27,30]. Together, these findings highlight that NET formation depends on the MPO-H_2_O_2_ oxidative burst pathway, consistent with other studies [12,13], which can be quantified using L-012.

In vivo imaging revealed increased ROS levels in both spontaneous and subcutaneous PDAC models compared to healthy tumor-free mice. Given the MPO-specificity of L-012 and luminol, our data suggest a substantial presence of MPO-activated neutrophils within PDAC tumors, aligning with previous studies demonstrating that neutrophils comprise an abundant portion of the PDAC tumor microenvironment [14,15,53]. Beyond neutrophils, other myeloid subsets such as monocytes may also contribute to these heightened ROS levels observed in PDAC. Similarly to our previous studies in subcutaneous PDAC models [31], increased ROS and NETs were observed in basal Gr1+ myeloid cells isolated from the spleen of KPC tumor-bearing mice, compared to healthy mice (Figure S4a,b). This effect was observed even without additional stimulation, suggesting that the presence of the tumor itself was sufficient to elevate ROS and NETs in splenic-derived myeloid cells, underscoring PDAC as a systemic disease. An extended correlation from a previous study done in our lab showed a strong correlation between basal ROS and NETs in these Gr1+ myeloid cells isolated from subcutaneous KPCY6419 tumor-bearing WT and *MPO^−/−^* mice (Figure S4c). As Gr1^+^ myeloid cells include the Ly6G^+^ neutrophil marker, these findings reinforce the connection between neutrophil ROS and NETs driven by PDAC.

Interestingly, our data suggest a bidirectional relationship between NETs and ROS in a subcutaneous PDAC model. *PAD4^−^/^−^* and HCQ-treated mice, both of which exhibit impaired NETosis, showed significantly reduced intratumoral ROS levels (Figure 3a,b and Figure S5) that were comparable to those seen in *MPO^−^/^−^* mice, which we previously reported had decreased NETs in neutrophils [31]. Further, our data showed no fold change in ROS levels following DNase treatment (Figure 3c), which degrades the NETs structure after their formation. This strongly implies that once NETs have been produced, their subsequent degradation does not lead to a reduction in ROS. Instead, it is the trapped enzyme activities during NET formation, rather than the presence of NET scaffolds or their breakdown, that are critical for the elevated ROS levels within the tumor microenvironment. Therefore, while L-012 can effectively detect ROS associated with ongoing NET formation, it cannot directly monitor NET degradation, as ROS levels remain unaffected following NET breakdown by DNase. Thus, in models where ROS production is dependent upon NETosis, our data suggest that L-012 and luminol bioluminescence serve as a reliable readout of NET formation. Our studies utilize *MPO^−^/^−^* and *PAD4^−^/^−^* mice because these models selectively disrupt the two principal neutrophil effector pathways most closely linked to NET biology: (1) the NADPH–MPO oxidative pathway and (2) PAD4-dependent chromatin decondensation. Using these strains allows us to modulate NET formation while preserving the presence and recruitment of neutrophils themselves, enabling a more direct assessment of the ROS–NET relationship in vivo. The marked reduction in tumor ROS observed in both *MPO^−^/^−^* and *PAD4^−^/^−^* mice, as well as following pharmacologic NET inhibition with HCQ, support the interpretation that neutrophils, and specifically NET-associated pathways, are major contributors to the elevated ROS detected in PDAC tumors. Previous studies have used fluorescent ROS probes, such as 2′,7′-dichlorodihydrofluorescein diacetate, cytochrome c reduction assays, and CellROX, to quantify ROS levels and oxidative stress in cells; however, these fluorescent probes have a few limitations, including a lower signal-to-noise ratio, dependence on excitation light, and susceptibility to photobleaching, which may reduce the reliability of fluorescent probes for capturing the rapid, high-flux ROS bursts characteristic of NETosis, particularly in in vivo settings. While fluorescent reporters targeting neutrophil elastase and cathepsin G have been used to detect NETosis [54,55], in vivo bioluminescence imaging offers substantially higher quantifiability due to its lower background signal and improved signal-to-noise ratio. Accordingly, although L-012 is not inherently specific to NET-derived oxidants, its high sensitivity and chemiluminescent properties make it particularly well suited for detecting the rapid, high-flux ROS burst that accompanies NET formation, thereby providing a powerful tool for monitoring NET dynamics in real time.

Extending our findings to clinical PDAC samples, we confirmed elevated ROS levels in ex vivo surgically resected tumor tissues compared to adjacent normal tissue (Figure 5a). It is essential to recognize that PDAC is a systemic disease with increased neutrophil infiltration documented even in non-tumor surrounding and distant tissues [53]. As a result, tumor-adjacent tissue may exhibit higher ROS levels than a normal healthy pancreas, likely reflecting the broader systemic influence of the tumor. Consistent with this, circulating neutrophils isolated from the blood of PDAC patients exhibited significantly higher ROS levels compared to those from patients with IPMN, a precancerous cystic neoplasm. This suggests that the elevated ROS in PDAC is more likely tumor-specific than a premalignant condition. Nearly 15–20% of PDACs are thought to arise from mucinous pancreatic cysts, the majority of which are IPMNs [56,57]. Early detection of PDAC significantly improves clinical outcomes, with localized disease showing a 5-year relative survival rate of 44% according to the American Cancer Society. Our findings demonstrate that blood-isolated neutrophils from PDAC patients exhibit elevated L-012 bioluminescence compared to those from IPMN patients, suggesting that this assay may serve as a low-cost, simple, and noninvasive tool for longitudinal monitoring of IPMN progression to PDAC. In addition, the correlation between neutrophil ROS levels and NET formation in patients mirrors the preclinical data, underscoring the translational relevance of these findings to human disease (Figure 5d). This correlation between ROS and NETs suggests that L-012 may have broader clinical applicability as an imaging probe for NETs beyond ROS-mediated inflammation alone. The L-012–based assay could be readily incorporated into clinical ex vivo workflows to screen for systemic or local inflammation by quantifying ROS or NET levels from patient blood or tissue samples. Because elevated ROS and NET formation have been linked to tumor progression and therapeutic resistance in PDAC [18,19,58], L-012 may help identify patients with highly inflammatory or NET-driven tumors and identify patients who could benefit from anti-inflammatory or anti-NET therapies. Notably, patients treated with HCQ showed a trend toward reduced neutrophil ROS and decreased serum levels of the NET marker (citH3), consistent with previous clinical observations (Figure 5c and Figure S6) [33]. L-012 imaging of blood isolated neutrophils may offer a strategy to monitor systemic NET inhibition levels in patients during HCQ treatment. More broadly, this assay may provide a useful approach for treatment monitoring across a range of ROS- or NET-targeted therapeutic interventions.

Given that the use of bioluminescence probes like L-012 is not clinically translatable for in vivo imaging in patients, our study successfully used [^18^F]4FN PET imaging to non-invasively visualize intratumoral ROS in a PDAC murine model. Previous studies have shown [^18^F]4FN PET selectivity for MPO-derived oxidative products, making it a valuable tool for assessing innate immune activation [30]. Importantly, [^18^F]4F PET imaging is currently undergoing evaluation in a phase I clinical trial (NCT05335811) to assess safety, biodistribution, and dosimetry profile, supporting its potential for clinical translation. Beyond the scope of this study, future studies will evaluate how [^18^F]4FN PET imaging may also be used as a non-invasive strategy to monitor PDAC development in high-risk IPMN patients. The increased PDAC tumor uptake of [^18^F]4FN, a functional analog of L012, along with the positive correlation of ROS and NETs in our study, further support its clinical application in tracking in vivo ROS/NETs in cancer.

While promising, several limitations should be acknowledged in this study. Our work primarily focused on MPO-dependent ROS and did not comprehensively explore the contributions of other upstream oxidative species such as NOX or reactive nitrogen species which may contribute to ROS production and NET formation. In addition to MPO and NOX, neutrophil-independent sources of ROS such as monocytes, macrophages, or tumor cells may also drive L-012 signal, potentially confounding the observed in vivo correlation between ROS levels and NET markers. Future studies should incorporate broader profiling of oxidative stress pathways, including assays for NOX, nitrogen species, and different cellular sources of ROS to better characterize the full spectrum of oxidative drivers. The ROS-NET correlations observed in human samples were neutrophils isolated from the peripheral blood of PDAC patients. Baseline neutrophil activation varies substantially across patients which may be affected by systemic inflammation, tumor burden, and treatment history. Larger patient cohorts, as well as studies incorporating tumor-infiltrating neutrophils, will be needed to strengthen and validate these correlations. While we noted a trend toward reduced neutrophil-derived ROS in HCQ-treated patients compared to those receiving neoadjuvant therapy, the difference did not reach statistical significance, likely reflecting limited sample size and highlighting the need for larger clinical datasets. Additionally, NET formation and ROS generation are not cancer-specific processes. Inflammatory conditions such as acute pancreatitis, chronic inflammation, and intra-abdominal infections can elevate ROS and NET levels independently of tumor biology, underscoring the need to evaluate these imaging approaches in broader inflammatory cohorts. As an initial step, we included IPMN samples, a clinically relevant non-malignant pancreatic condition, and observed that L-012 bioluminescence was specifically elevated in PDAC patients. Nonetheless, prospective studies across diverse inflammatory pathologies will be essential to define specificity and avoid false-positive interpretations in the clinic. Postoperative or treatment-related inflammation may confound ROS-based imaging, emphasizing the importance of appropriate timing and clinical context when interpreting L-012 signals. Another limitation is that [^18^F]4FN PET imaging was evaluated only in a subcutaneous PDAC model. To better assess the translational potential of this probe, future studies will evaluate the broad range of preclinical models, including orthotopic and spontaneous PDAC models that better recapitulate the native PDAC tumor microenvironment to evaluate the utility of [^18^F]4FN PET imaging to monitor inflammation in PDAC. Together, these limitations highlight that the present study is exploratory and correlative. Additional mechanistic work, validation in inflammatory disease controls, and prospective clinical studies will be required to establish the specificity, safety, and interpretive framework necessary for clinical application.

Overall, our study demonstrates a positive correlation between ROS levels and NET production in both PDAC murine models and blood-isolated PDAC patient neutrophils, suggesting the potential use of L-012 as a surrogate marker of NETosis. The validation of [^18^F]4FN PET imaging as a tool for in vivo ROS detection holds translational potential for patient monitoring of PDAC progression and treatment efficacy, given the known correlation of increased ROS/NETs with poor outcomes and treatment responses [18,19,58]. Finally, these imaging modalities open new avenues for visualizing and assessing the therapeutic effects of various treatments involving neutrophil-targeted ROS and NET inhibitors in vivo, not only in cancer but also in other inflammation-associated diseases.

## Figures and Tables

**Table 1:** Patient demographic and clinical characteristics.

	IPMN (N = 5)	PDAC (N = 49)
Mean Age (SD)	63.4 (7.5)	65.9 (12.6)
Gender, n (%)	Male, 2 (40)	Male, 24 (48.98)
	Female, 3 (60)	Female, 25 (51.02)
Stage, n (%)	N/A	
I		15 (30.61)
II		21 (42.86)
III		10 (20.41)
IV		2 (4.08)
NS		1 (2.04)
Treatment, n (%)	N/A	
Untreated		15 (30.61)
FOLFIRINOX		20 (40.82)
Gemcitabine		6 (12.24)
HCQ + FOLFIRINOX		7 (14.29)
HCQ + Gemcitabine		1 (2.04)

N/A, not applicable; NS, non-specified.

## Data Availability

The original contributions presented in this study are included in the article/Supplementary Material. Further inquiries can be directed to the corresponding author.

## Supplementary Materials

The following supporting information can be downloaded at: https://www.mdpi.com/article/10.3390/antiox14121473/s1, Figure S1: L-012 specificity for neutrophil ROS; Figure S2: Luminol specificity for neutrophil MPO-derived ROS and its correlation with NETs formation; Figure S3: In vivo MPO activity in murine PDAC models using luminol; Figure S4: Correlation of ROS with NETs in Gr1^+^ myeloid cells from tumor-bearing mice; Figure S5: In vivo targeting of NET formation decreases ROS level assessed by luminol; Figure S6: Quantification of serum CitH3 ELISA in PDAC patients treated with HCQ compared to the neoadjuvant (Neo) group.

## Abbreviations

The following abbreviations are used in this manuscript:

CPTIO	2-(4-Carboxyphenyl)-4,4,5,5-tetramethylimidazoline-1-oxyl-3-oxide
DPI	Diphenyleneiodonium
[^18^F]4FN	4-[^18^F]fluoro-1-naphthol
HCQ	Hydroxychloroquine
IPMN	Intraductal papillary mucinous neoplasm
MPO	Myeloperoxidase
Neo	Neoadjuvant
NETs	Neutrophil extracellular traps
NOX	NADPH oxidases
PAD4	Peptidyl arginine deiminase 4
PAF	Platelet-Activating Factor-16
PDAC	Pancreatic ductal adenocarcinoma
PET	Positron emission tomography
PMA	Phorbol 12-myristate 13-acetate
ROS	Reactive oxygen species
Verd	Verdiperstat
