# Supplementary material for: Imaging Reactive Oxygen Species with L-012 Reveals Neutrophil Extracellular Trap Formation in Pancreatic Ductal Adenocarcinoma
Source: Antioxidants (Basel). 2025 Dec 8;14(12):1473. doi: 10.3390/antiox14121473 (PMC12729264; doi:10.3390/antiox14121473)
Supplement: Supplementary file 1 [file antioxidants-14-01473-s001.zip › antioxidants-3986354-supplementary.pdf]

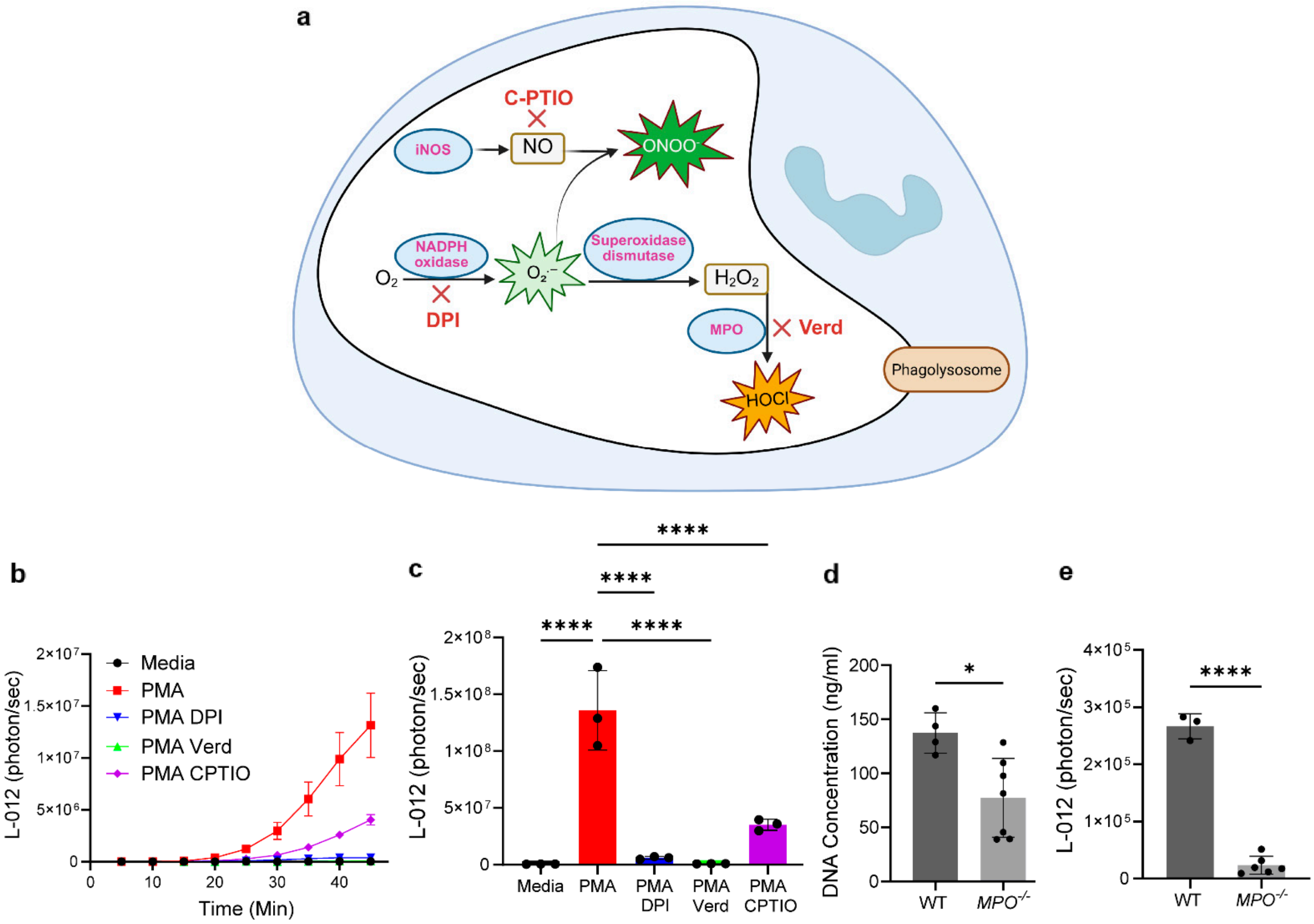

Figure S1. L-012 specificity for neutrophil ROS **(a)** Schematic illustration of the respiratory burst pathways in neutrophil with inhibitors targeting production of specific ROS **(b, c)** HL60 cells differentiated into 'neutrophil-like' cells were stimulated with PMA. Stimulated cells were incubated with DPI, Verd, CPTIO, and then imaged using the L-012 reporter; unstimulated, cells alone (media) were used as a control. The data represent the time kinetics and the area under the curve value. Quantification of **(d)** NET formation and **(e)** ROS production in unstimulated (basal) neutrophils isolated from bone marrow of WT and *MPO*$^{-/-}$ mice. Data are representative of n = 3 – 7 independent experiments and shown as mean ± SD. Statistical significance was determined using one-way ANOVA followed by Tukey's multiple comparison test and unpaired student *t*-test, *p<0.05, ****p<0.0001.

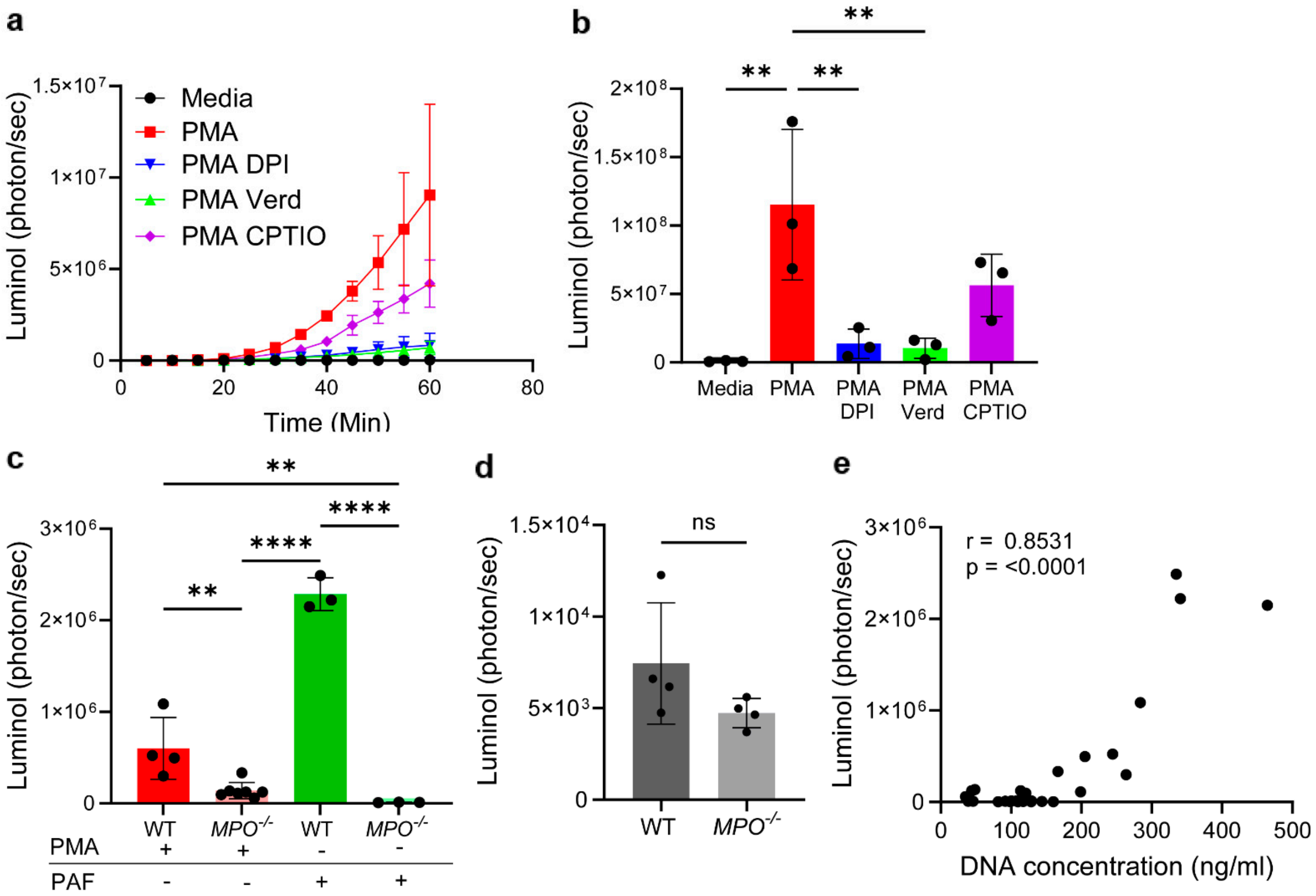

Figure S2. Luminol specificity for neutrophil MPO-derived ROS and its correlation with NETs formation. **(a, b)** HL60 cells differentiated into 'neutrophil-like' cells were stimulated with PMA. Stimulated cells were incubated with DPI, Verd CPTIO, and then imaged using the luminol reporter; unstimulated, cells alone (media) were used as a control. The data represent the time kinetics and the area under the curve value. Quantification of luminol-based MPO activity in **(c)** PMA and PAF stimulated neutrophils **(d)** unstimulated (basal) neutrophils isolated from bone marrow of WT and *MPO*$^{-/-}$ mice. **(e)** Correlation analysis between luminol BLI signal (MPO activity) and NET formation. Data are representative of at least three independent experiments and shown as mean ± SD. Statistical significance was determined using one-way ANOVA followed by Tukey's multiple comparison test and unpaired student *t*-test, **p<0.01, ****p<0.0001. Correlation analysis was performed using Pearson's correlation coefficient (r).

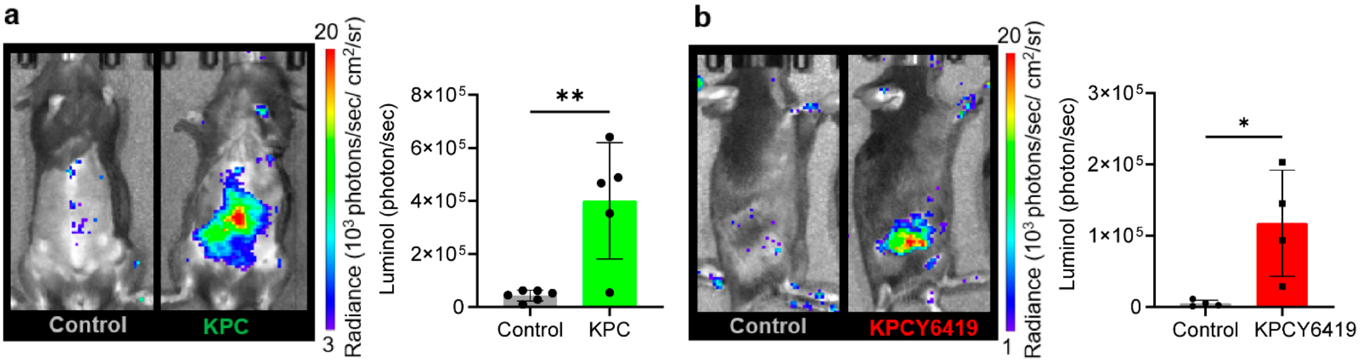

Figure S3. *In vivo* MPO activity in murine PDAC models using luminol. Representative images and quantification of luminol bioluminescence in **(a)** spontaneous KPC (week 12 – 17) and **(b)** subcutaneous KPCY6419 PDAC tumors at endpoint (day 24 post tumor cell injection) compared to healthy tumor-free controls (WT). Data shown as mean ± SD; n=3 – 5 mice/group. Statistical analysis was performed using an unpaired student *t*-test, *p<0.05, **p<0.01.

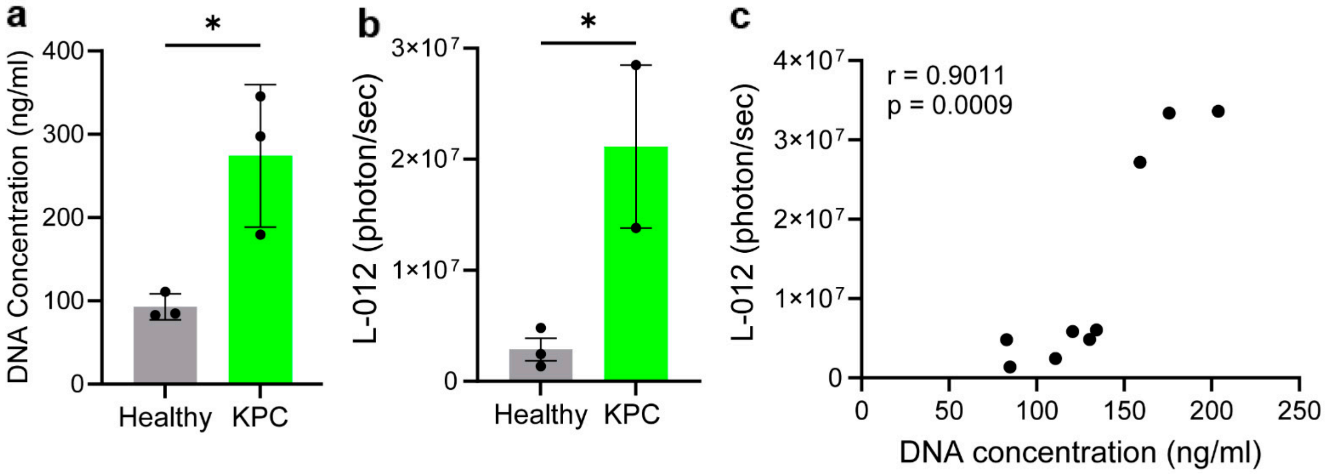

Figure S4. Correlation of ROS with NETs in Gr1+ myeloid cells from tumor-bearing mice. Quantification of **(a)** NETs and **(b)** ROS production in Gr1+ myeloid cells isolated from the spleen of tumor-bearing KPC and healthy (tumor-free WT) mice. **(c)** Correlation analysis between L-012 BLI signal (ROS levels) and NET formation in Gr1+ splenic myeloid cells isolated from healthy (tumor-free WT) and subcutaneous KPCY6419 PDAC tumor-bearing WT and *MPO*$^{-/-}$ mice using previously published data from our lab [40]. Data shown as mean ± SD; n = 2 – 3 mice/group. Statistical analysis was performed using an unpaired student *t*-test, *$p<0.05$. Correlation analysis was performed using a Pearson's correlation coefficient (r).

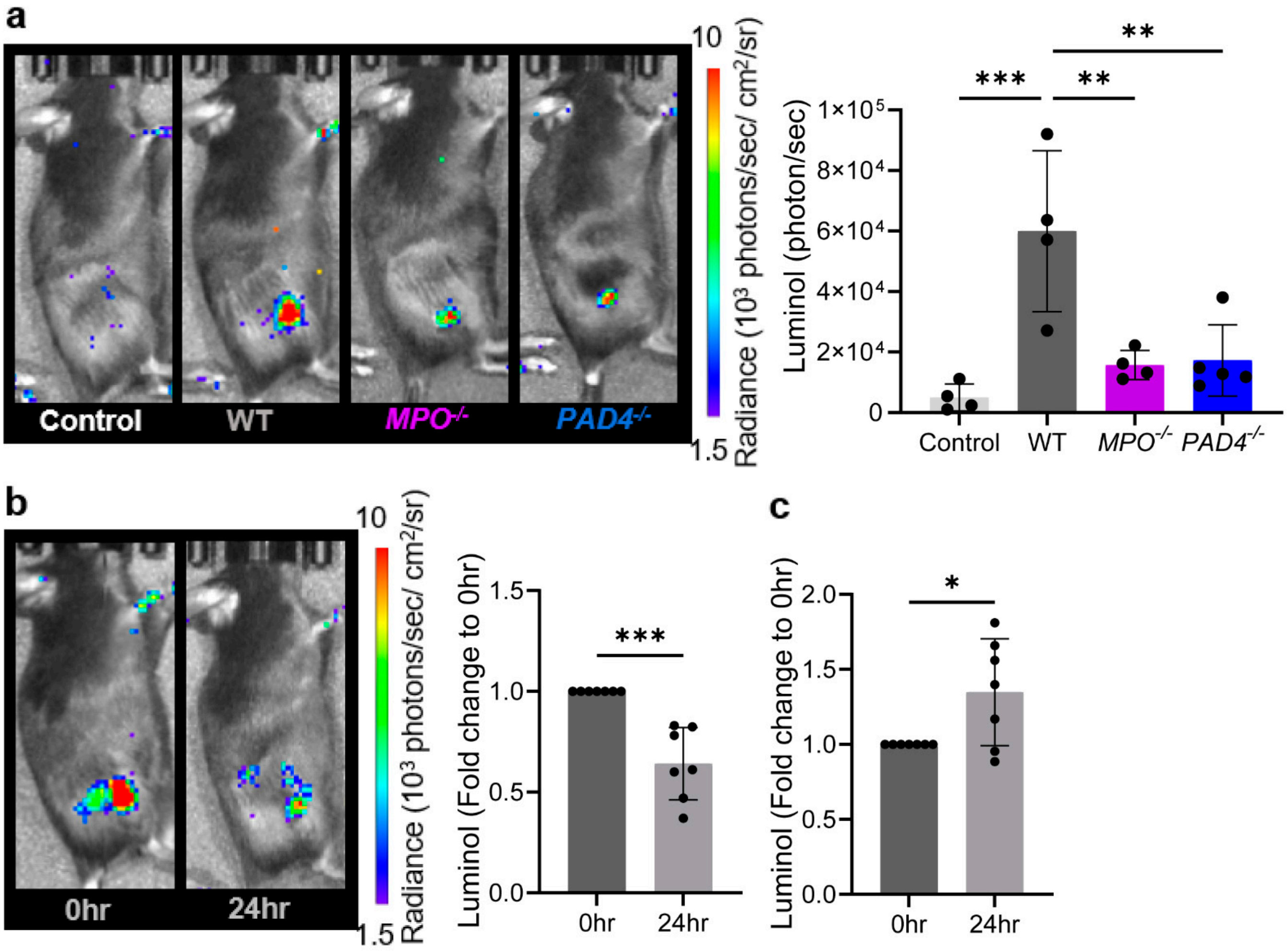

Figure S5. *In vivo* targeting of NET formation decreases ROS level assessed by luminol. **(a)** Representative bioluminescence images and corresponding quantification of MPO activity using luminol in **(a)** healthy WT tumor-free controls and WT, *MPO*$^{-/-}$, *PAD4*$^{-/-}$ subcutaneous KPCY6419 tumor-bearing mice at day 17 post tumor cell injection (control mice are the same dataset from figure S3b), **(b)** pre (0 h) and 24 h post (24 h) HCQ treatment at days 14 and 17 post tumor injection and **(c)** pre (0 h) and 24 h post (24 h) DNase treatment at days 14 and 22 post tumor injection in KPYC6419 tumor-bearing WT mice. WT data shown in panel a (WT) and panel b (0 h) are derived from the same cohort of mice at day 17 post tumor injection. Data shown as mean ± SD; n = 3 – 7 mice/group. Statistical analysis was performed using one-way ANOVA followed by Tukey's multiple comparison test and an unpaired student *t*-test, *p<0.05, **p<0.01,***p<0.001.

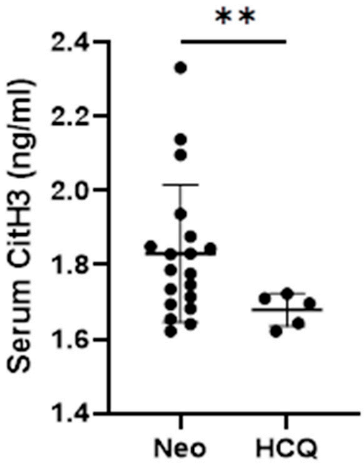

Figure S6. Quantification of serum CitH3 ELISA in PDAC patients treated with HCQ compared to the neoadjuvant (Neo) group. Statistical analysis was performed using an unpaired student *t*-test, **p<0.01.
